# Changes in climate extreme indices and agricultural drought monitoring in the semi-arid areas of Borana zone, southern Ethiopia

**DOI:** 10.1016/j.heliyon.2025.e42041

**Published:** 2025-01-19

**Authors:** Girma Asefa Bogale, Asfaw Kebede Kassa, Mengistu Mengesha Maja

**Affiliations:** a School of Natural Resources Management and Environmental Sciences, College of Agriculture and Environmental Sciences, Haramaya University, P.O. Box 138, Dire Dawa, Ethiopia; b School of Water Resource and Environment Engineering, Haramaya University, P.O. Box 138, Dire Dawa, Ethiopia

**Keywords:** Adaptation option, Drought indices, Extreme indices, Rainfall indices, Borana zone

## Abstract

Climate variability and extremes negatively impact on agricultural productivity and production in arid and semi-arid areas of Ethiopia. This study evaluated changes in climate extreme indices in the semi-arid areas of Borana Zone. Data of daily rainfall, maximum and minimum temperature from 1993 to 2022 were acquired from the National Meteorological Institute of Ethiopia and Climate Hazards Group InfraRed Precipitation with Station data (CHIRPS). R-package for climate extreme indices (RClimDex), Xlstat 2019, OriginPro, and ArcGIS software were used as data analysis tools to determine climate extreme indices, trends, and drought anomalies in the study area. Spatial and temporal variations in rainfall, temperature, and vegetation indices in the semi-arid Borana zone were analyzed using Google Earth Engine (GEE). The results revealed a decrease in the frequency of consecutive wet days (CWD), maximum 1-day precipitation amount (Rx1days), maximum 5-day precipitation amount (Rx5days), and very wet days (R95p) in Dire, Guchi, Wachele, and Moyale districts of Borana zone. Annual and monthly total precipitation (PRCPTOT) also decreased in Dhaso, Dilo, Dubluk, Guchi, Moyale, and Tatlate districts of the semi-arid areas of the zone over the study years. Warm spell duration index (WSDI), monthly or annual maximum of daily minimum temperature (TNx), warm nights (TN90p), and warm days (TX90p) showed an increase in the study area. However, standardized precipitation indices of R10, R20, R95p, and R99p indicated moderate drought across the studied districts of Borana zone. The Vegetation Condition Index (VCI) showed higher drought severity and spatial distribution in Dilo and Yabelo, whereas Temperature Condition Index (TCI) detected in Dire, Dubluk and Yabelo areas indicated drought stress due to moisture and thermal stress. Moisture-stress and thermal-stress play role in drought formation, whereas, drought severity is determined by moisture availability, which is dependent on rainfall. We demonstrate that the study area experienced moderate to severe drought frequent over the study years, and multiple satellite-based drought indices are necessary for better early warning and assessment of agricultural drought in the study area. Further studies that consider socioeconomic aspects of the farmers in the area are necessary to better understand the impacts of climate extremes on smallholder farmers, and devise location-specific adaptation strategies.

## Graphical abstract

Fig. 5. Graphical abstract of the research study.

**Figure:**
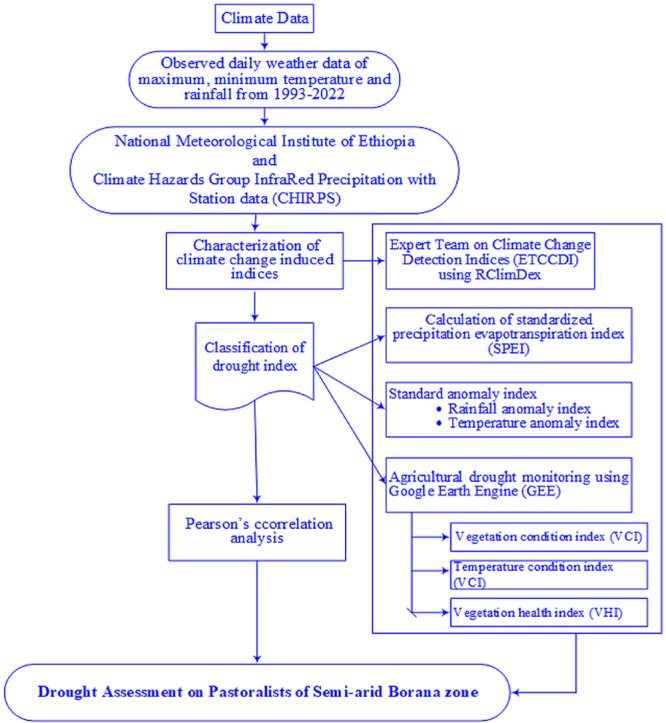

## 1 Introduction

Climate extremes have become more frequent, intense and had increased spatial coverage in recent decades due to climate change [1]. These extreme events individually or in combination can have profound impacts on agricultural production, economy and the environment [2,3]. Major climate extremes including drought, heat stresses and highly erratic rainfall conditions lead to changes in soil environment, which affects agricultural production and livelihoods [1]. Weather and climate extremes and their impacts are intensifying worldwide, but the negative impacts are more severe in poorer regions of the world such as the sub Saharan Africa (SSA) due to various factors including dependence on rain fed agriculture, high population pressure and limited adaptive capacity [4].

Southeast Asia and SSA are among the most vulnerable regions to climate extreme events due to high dependence on rain fed agriculture and limited adaptive capacity [4]. East Africa in particular faces frequent droughts and erratic rainfall which left millions of people in need of humanitarian assistance [5,6]. The food demand of over 300 million people living in East Africa is largely fulfilled by poor smallholder farmers with high vulnerability and limited adaptation capacity to climate extremes [7]. For example, as frequency, severity and area affected by climate extremes increase, more people will be food insecure in Ethiopia in the near future (FDRE, 2015). East Africa has experienced positive trends in the maximum and minimum extreme temperatures in the last few decades with a measurably notable increase during the 1980s (Tmax and T-min) [8,9]. Understanding the pattern of climate extremes is paramount to devise adaptation and mitigation strategies to offset the adverse impacts of these extremes.

In order to detect changes in the behaviour of climate extremes, the Expert Team on Climate Change Detection and Indices (ETCCDI), supported by the Joint Technical Commission for Oceanography and Marine Meteorology (JCOMM), the Climate Predictability and Variability (CLIVAR) project, and the Commission for Climatology (CCI) of the World Meteorological Organisation (WMO), developed a set of 27 indices (16 related to temperature and 11 related to precipitation) [10]. This set of indices was created to track changes in climate extremes globally in an identical manner in order to establish a common global baseline, which enables monitoring the occurrence of climate extremes and climate prediction [11].

Drought, a hydrometeorological phenomenon representing water shortages relative to normal conditions, is a key climate extreme that causes heavy losses in agricultural sector especially in semi-arid areas [12]. Its occurrence, severity and frequency are increasing as a result of rising temperature in many tropical areas with serious effect on food security, health and displace many from their homes. Agricultural drought is related to shortage of soil moisture in relation to climate and water scarcity for agricultural crop and livestock productivity as well as financial profitability. For meteorological and agricultural reasons, drought is tracked and assessed based on ground-based point observation data and spatial interpolation techniques [13]. However, ground-based observation data has less information than anticipated when it comes to continuous tracking of precipitation, soil surface temperature, wind speed, atmospheric water vapour, and relative humidity. Furthermore, the continuity of the current observation is insufficient to disclose the temporal and spatial variability of data connected to the drought [14]. Consequently, this study concentrates on remote sensing datasets for vegetation and climate factors to determine indices that help to identify drought characteristics. Drought severity, extensiveness and other extreme events can be closely monitored using geographical information systems methods and softwares.

Google Earth Engine (GEE) is a platform that provides access to large geospatial data and satellite imagery. It helps environmental monitoring for sizable regions, and its potential for analysis of geospatial data helps to address challenges associated with scarcity and discontinuity of ground-based data on climate extremes. The GEE machine learning methods make use of the Python and JavaScript programming languages in addition to high-speed parallel processing through the usage of Google's computing infrastructure. Therefore, large-scale geographic data analysis and visualization are made possible by the GEE [15]. Satellite based indices such as Normalized Difference Vegetation Index (NDVI) and Vegetation conditions (VC) can help characterization of drought and its spatial extent [16]. The NDVI in particular is an efficient and commonly employed vegetation index that is closely linked to drought conditions [17]. It is an indicator of plant density and health, with high values indicating healthier and more dense vegetation while low values imply sparse or no vegetation status of an area [17].

Drought is a substantial threat of crop and livestock productivity particularly in Ethiopia where rainfall-dependent agriculture is the backbone of the economy [18], thus monitoring of drought and other climate extremes is crucial. In recent decades, the southern and eastern dry lands of Ethiopia have been hit hard by rising temperature, rainfall uncertainties and other climate extremes. For instance, Ayal et al. [19] noted serious moisture stress in Borana zone due to decrease in the number of rainy days, volume of rainfall, rising temperature, which adversely affected the health of livestock, crop production and livelihoods. A similar study by Worku et al. [20] revealed a highly variable rainfall and rising temperature in semi-arid Borana zone at temporal and spatial scales. In many arid and semi-arid parts of Ethiopia including Borana and Guji zones, annual total precipitation (PRCPTOT), the number of heavy precipitation days (R10mm), and consecutive wet days (CWD) are decreasing [[20], [21], [22], [23]].The uncertain rainfall and rising temperature facilitate favourable condition for parasites and pathogens thereby leading to livestock diseases and reduced productivity, and lower cattle market prices [4,19,24].

Drought can be more accurately monitored using remotely sensed vegetation indices such as NDVI and VCI as these indices capture the spatial and temporal variations in water usage [25]. NDVI monitors drought over time, which helps to highlight the detrimental effects of moisture economy on vegetation for long-term [26]. This study attempts to fill the lack of monitoring of climate extremes using remote sensing techniques in Sothern Ethiopia. Therefore, the objective this study was to evaluate the trends and variability of rainfall and temperature of long-term data, to analyse the impacts climate change on pastoralist areas, and to monitor agricultural drought in the semi-arid areas of Borana zone, Southern Ethiopia.

## 2 Research methodology

### 2.1 Description of the study area

**Fig. 1:**
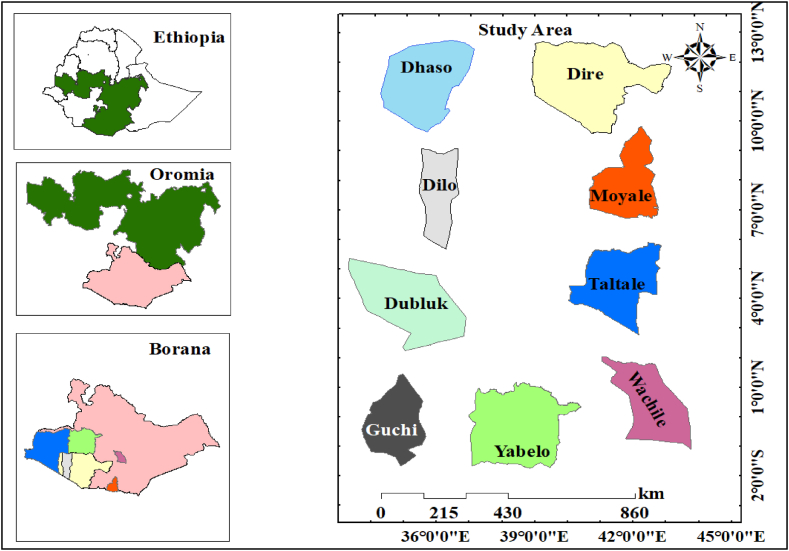
Geographical location of Borana Zone.

This study was carried out in nine districts, namely (Dhaso, Dilo, Dire Dubluk, Guchi, Moyale, Taltale, Wachile, and Yabelo) of Borana Zone, Oromia Region, Ethiopia (Fig. 1). Borana zone is located about 600 kms South of Addis Ababa, the capital city of Ethiopia. According to the Central Statistics Agency (CSA) projection, the area has a total population of 503,877, and approximately 89 % of the population lives in the rural pastoralist areas [27]. The Borana zone covers an area of approximately 95,000 km^2^, with an overall population density of six inhabitants per square kilometre. In Ethiopia, pastoralist communities represent 12 % of the total population and they basically reside in the arid and semi-arid lowland areas of the country which are normally vulnerable to rainfall variability [28]. The Borana rangelands cover approximately 19,000 km^2^ and the area is characterized by semi-arid savannah landscape, marked by gently sloping lowlands and flood plains that are mainly covered in bushes and grass [29]. The geology is composed of sedimentary and volcanic layers on top of a crystalline basement.

### 2.2 Climate and agroecological conditions

The climate of the country is varied and covers from semi-arid deserts to humid and temperature climate. In general, the southwest highlands receive the most rainfall (>2000 mm) and its south-eastern as well as north-eastern lowlands of the country receive the least (<300 mm) rainfall across the area. Ethiopia has various agro-ecological conditions, namely; *bereha* (hot arid), *kolla* (warm semi-arid), *dega* (cool and humid), *weyna-dega* (cool sub-humid), and *wurch* (cool and moist) [30], which enable the production of different crops and livestock.).

**Fig. 2:**
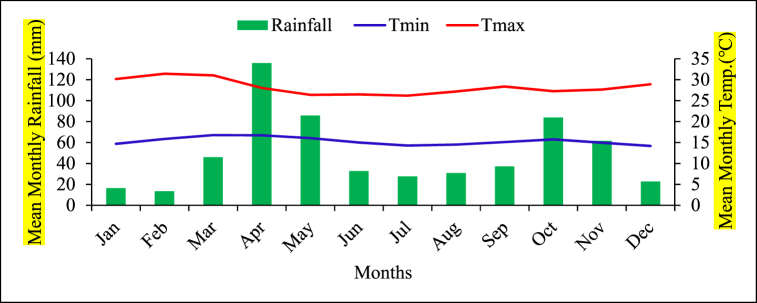
The general climate information in semi-arid Borana zone (1993–2022).

Semi-arid areas of Borana zone have a hot climate with average annual temperature that ranges from 18 to 29 °C. The area experiences mean maximum and minimum temperatures in the months of February (31.42^o^C) and December (14.17 °C) (Fig. 2). The area is categorised under a semi-arid climate and a bimodal precipitation distribution, by a long stormy season beginning on March to May then brief downpours from September to October.

### 2.3 Socio-economic activities

Livestock are the major sources of livelihood for the Borana pastoralists. Borana zone is an epicentre of livestock production, and famers use income from livestock sale to pay for food and other necessities for their families. Additionally, the percentage of homes in the area that consumed large and small livestock was roughly 71 % and 79 %, respectively. Similarly, a significant portion of households (79 %) produced and consumed milk and other livestock products, and 40 % marketed livestock goods, around 60 % of the respondents reported selling large livestock (cattle), and 70 % sold small livestock (sheep, goat, and chickens) [31].

### 2.4 Methods of data collection and sources

Daily historical climate data was collected from the National Meteorological Institute of Ethiopia for the period of 1993–2022. In addition, Climate Hazards Group Infra-Red Precipitation with stations (CHIRPS) satellite rainfall data was used because most rainfall data from *in-situ* meteorological stations had short period records and a large percentage of missing data problem [[32], [33], [34]]. The CHIRPS dataset is a quasi-global dataset covering the area between 50^o^ N and 50^o^ S available from 1981 to present-day at 0.05^o^ spatial resolution (∼5.3 km) and it is produced using multiple data sources [35].

### 2.5 Methods of research data analysis and interpretation

#### 2.5.1 Data quality control

In this study, data quality control was done by R_program version 4.4.1 and multiple imputation by chained equations (MICE) [36] to resolve any missing estimation data acquired from the National Meteorological Institute of Ethiopia. Because MICE can generate multiple imputations to impute missing values in the original dataset, as demonstrated in the works of Zhang [37] and Siabi et al. [38]. According to Abdullah et al. (2022), MICE technique is an appropriate method for data quality control for climate analysis. In MICE analysis, each dataset used in the study had fewer than 2 % of missing data. It is noteworthy that locations that are especially vulnerable to climate variability frequently have major data gaps as noted by Wilby et al. [39], and the quality of climate data is a key concern in developing countries [38].

#### 2.5.2 Data homogeneity test

Unusual values of daily rainfall, maximum, and minimum temperature data for each station were identified using the RClimDex software version 1.0. In addition to identifying rainfall levels less than zero, the analysis required determining whether there were any instances where the daily Tmin was equal to or greater than Tmax. Furthermore, outliers of each dataset were found by applying a four-standard deviation criterion [40].

#### 2.5.3 Trend analysis

Trend analysis of extreme rainfall and temperature indices was carried out by the Mann-Kendall (MK) test [41,42]. The magnitudes of change evaluated using Sen's slope estimator [43].[44]. The degree and significance of the observed changes, whether increasing or decreasing were indicated using the Kendall tau statistics and the corresponding *p*-values at 95 % and 99 % confidence levels. Trend analysis is a non-parameters test that looks intended for a trend in a time series without indicating whether the trend is linear or non-linear [45]. The ZM test statistic “S” is calculated based on [41,45,46] using the formula in Equation (1),

(1) $$S=\sum_{i=1}^{n-1}\sum_{j=i+1}^{n}\mathrm{sgn}\;\left(\text{xj}-\text{xi}\right)$$

Where S is the Mann-Kendal's test statistics; xi and xj was the sequential data values of the.

time series in the years i and j (j > i) and N is the length of the time series. A positive S value

indicates an increasing trend and a negative value indicates a decreasing trend in the data

series. The sign function is given in equation (2):

(2) $$\mathrm{sgn}\;\left(\text{xj}-\text{xi}\right)=\left\{ \begin{array}{l} +1\;\text{if}\;\left(\text{xj}-\text{xi}\right)>0\\ 0\;\text{if}\;\left(\text{xj}-\text{xi}\right)=0\\ -1\;\text{if}\;\left(\text{xj}-\text{xi}\right)<0 \end{array}\right.$$

The variance of S, for the situation where there may be ties (that is equal values) in the X values, is given in equation (3):

(3) $$\text{Var}\left(s\right)=\frac{1}{18}[(N\left(N-1\right)-\sum_{i=1}^{m}t_{i}\left(t_{i}-1\right)\left(2t_{i}+5\right)]$$

Where, m is the number of tied groups in the data set and t_i_ is the number of data points in the ith tied group.

For n larger than 10, ZMK approximates the standard normal distribution [47,48] and given as follows in equation (4):

(4) $$Z=\left\{ \begin{array}{l} \frac{s-1}{\delta }\;if\;s>0\\ 0\;if\;s=0\\ \frac{s+1}{\delta }\;if\;s\;<0 \end{array}\right.$$

Where, S is variance. The presence of a statistically significant trend is evaluated using the ZMK value. Z1-α/2 is the critical value of ZMK from the standard normal table, example, for 5 % and 1 % significance levels, the value of Z1-α/2 is 1.96 and 2.33, respectively.

The Sen's estimator of slope was applied in cases where the trend is assumed to be linear, depicting the quantification of changes per unit time. This method could be used with missing data and remain unaffected by outliers or gross errors [49]. The slope (change per unit time) was estimated following the procedure [43].

### 2.6 Calculation of Expert Team on Climate Change Detection and Indices (ETCCDI)

Expert Team on Climate Change Detection and Indices (ETCCDI) developed a collection of 27 indices to describe instances of excessive rainfall and temperature. Based on the ETCCDI framework, thirteen (13) extremes of temperature and ten (10) rainfall indices were selected for the research conducted in the study area (Table 1). RClimDex1.1 program that was created [40] was used to compute these indices. In this regard, daily recorded precipitation, maximum, and minimum temperature data from the baseline period of 1993–2022 were used in the computations for each meteorological station.

**Table 1:** Description and definition of climate indices used in the analysis.

Types	Indices	Description	Definition	Units
	TR20	Tropical nights	The annual count of days when daily minimum temperature is greater than 20^o^C	Days
	CSDI	Cold spell duration index	A sequence of 6 or more days where daily minimum temperature is below the 10th percentile	Days
	WSDI	Warm spell duration index	A sequence of 6 or more days where daily maximum temperature is exceeds the 90th percentile	Days
Temperature	SU25	Summer days	The annual count of days where daily maximum temperature exceeds 25 °C	Days
indices	TNx	Max Tmin	Compute monthly or annual maximum of daily minimum temperature	^o^C
	TNn	Min Tmin	Compute monthly or annual minimum of daily minimum temperature	^o^C
	TN10p	Cool nights	Compute monthly or annual proportion of minimum temperature below 10th percentile	Days
	TN90p	Warm nights	Computes monthly or annual maximum of daily maximum temperature	^o^C
	TXx	Max Tmax	Computes monthly or annual maximum of daily maximum temperature	^o^C
	TXn	Min Tmax	Computes monthly or annual minimum of daily maximum temperature	^o^C
	TX10p	Cool days	Computes monthly or annual proportion of maximum temperature below 10th percentile	^o^C
	TX90p	Warm days	Computes monthly or annual proportion of maximum temperature above 90th percentile	Days
	DTR	Diurnal temperature range	Computes the mean daily diurnal temperature range. The frequency of observation can either be monthly or annual	^o^C

	R10	Number of heavy precipitation days	Annual count when precipitation ≥ 10 mm	Days
	R20	Number of very heavy precipitation days	Computes the annual count of days where daily precipitation is more than 20 mm per day	Days
Rainfall indices	SDII	Simple precipitation intensity index	Computes the annual sum of precipitation in wet days (days with precipitation over 1 mm) during the year divided by the number of wet days in the years	mm
	CDD	Consecutive dry days	Maximum number of days when precipitation is less than 1 mm	Days
	CWD	Consecutive wet days	Maximum number of days when precipitation is more than 1 mm	Days
	RX1day	Max 1-day precipitation amount	Computes monthly maximum 1-day precipitation	mm
	RX5day	Max 5-day precipitation amount	Computes monthly maximum consecutive 5-day precipitation	mm
	R95p	Very wet days	Computes the annual sum of precipitation in days where daily precipitation exceeds the 95th percentile	mm
	R99p	Extremely wet days	Computes the annual sum of precipitation in days where daily precipitation exceeds the 99th percentile	mm
	PRCPTOT	Annual total wet-day precipitation	Computes the annual sum of precipitation in wet days (days where precipitation is at least 1 mm)	mm

### 2.7 Pearson's correlation analysis

This method of statistical analysis looks at how strongly two variables are related to one another. It is used to look at the correlation between two quantitative variables and is denoted by the symbol r. The following equation is used to get the correlation coefficient [50], equation (5):

(5) $$r=\frac{n\sum XY-\sum X\sum Y}{\sqrt{n\sum X^{2}-{\left(\sum X\right)}^{2}}\sqrt{n\sum Y^{2}-{\left(\sum Y\right)}^{2}}}$$

Where, if r = 1, and −1 the correlation is said to be perfect positive, and negative respectively, while if r = 0, the variables X and Y are said to be uncorrelated [51].

### 2.8 Standardized Precipitation-Evapotranspiration Index (SPEI)

Standardized Precipitation-Evapotranspiration Index uses the basis of standardized precipitation index (SPI), allowing the index to account for the effect of temperature on drought development through a basic water balance calculation. SPEI features an intensity scale that determines positive and negative values, indicating wet and dry events [52]. For this analysis, drought frequency was analyzed by dividing the number of years of drought occurrence by the total number of years and multiplying by 100 % based on the SPEI-12 index value classification [53,54]. In this study, the average anomalies of precipitation and temperature were assessed in the agroecological zones of semi-arid Borana pastoralists. The SPEI values are calculated using equation (6) and the SPEI values and classes are given in Table 2.

(6) $$x=\frac{X-\bar{X}}{\delta }$$

Where, x is the recorded values, $\bar{x}$ is the mean of the data and ***σ*** is the standard deviation of the data

**Table 2:** Drought classification based on SPEI classes.

SPEI value	Classes
>2	Extremely wet
1.5 to 1.99	Severe wet
1 to 1.49	Moderately wet
0.99 to −0.99	No drought
−1 to −1.49	Moderately drought
−1.5 to −1.99	Severe drought
< −2	Extremely drought

### 2.9 Calculation of remote sensing four drought indices (NDVI, CVI, TCI and VHI)

**Fig. 3:**
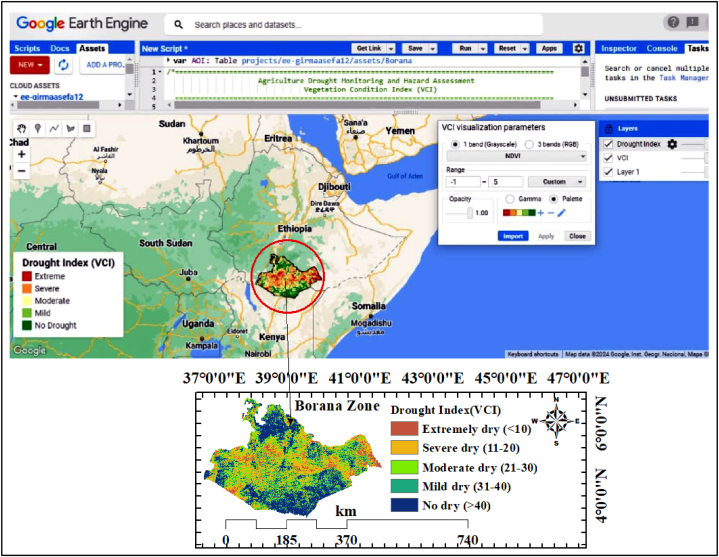
Drought monitoring at Borana zone using Google Earth Engine.

Google Earth Engine (GEE) is a cloud-based platform powered by Python API and Java Scripting [55]. GEE is a useful tool for managing large geodata sets. It is faster than traditional software and can reduce processing times in half. Researchers without any coding knowledge can easily and swiftly make use of the many datasets that GEE has made accessible through its repository. Borana zone was extracted from Google Earth Engine with acknowledged agricultural drought monitoring and classification (Fig. 3).

The maximum amount of vegetation grows annually under ideal weather conditions as these circumstances encourage the effective use of ecosystem resources such as a rise in the rate of soil nutrients absorbed [56]. On the other hand, minimum vegetation amounts occur in years of exceptionally adverse weather conditions, primarily dry and hot, which inhibit vegetation growth both directly and indirectly by decreasing the rate at which environment resources are used (e.g., in the drought years the amount of soil nutrient uptake is significantly affected by water shortage) [56]. The NDVI is a widely used metric for Vegetation monitoring, forecasting, and crop yield evaluation [[57], [58], [59]]. The vegetation condition index (VCI) was developed [60] to study the global responses of plants to drought. Equations presented in Table 3 are used to further define the vegetation health index (VHI), temperature condition index (TCI), and vegetation condition index (VCI) [61]. VCI rescales vegetation dynamics between 0 and 100, where smaller VCI values indicate worse vegetation growth and higher degree of vegetation dryness [58,60].

**Table 3:** Equation of tested four remote sensing indices.

No.	Remote Sensing Based Indices	Equation	Reference
1	Normalized Difference Vegetation Index	$NDVI=\frac{NIR-RED}{NIR+RED}$	[62]
2	Vegetation Condition index	$VCI=\frac{\left(NDVI-\text{NDVImin}\right)}{\left(NDV\text{Im}ax+NDV\text{Im}in\right)}*100$	(F. N. [63])
3	Temperature Condition Index	$TCI=\frac{T\;\max-\text{Tc}}{T\;\max-\text{Tmin}-}*100$	(F. N. [63])
4	Vegetation Health Index	VHI=0.5∗VCI+0.5∗TCI	[63]

NDVI, NDVImin, and NDVImax are the seasonal average of the smoothed weekly NDVI, its multiyear absolute minimum and its maximum, respectively, NIR; Near Infrared Reflectance, RED; Reflectance in the red band. Tc, Tmin, and Tmax are similar values for land surface temperature in Celsius.

Healthy plants have high VCI levels when they are not under stress. Temperature condition index (TCI) enables assessing the stress caused by temperature and wetness. In conjunction with VCI and NDVI, it helps to monitor agricultural drought. A low TCI values signify hot weather and stress on vegetation caused by high temperatures, often associated with drought conditions [64]. The drought classification and the values of VCI, TCI and VHI are given in Table 4.

**Table 4:** Drought classification in terms of the VCI, TCI and VHI values.

Drought	Values
Extreme dry	<10
Severe dry	11–20
Moderate dry	21–30
Mild dry	31–40
No dry	>40

In this study, water, snow or ice, urban areas and bare land were excluded from the final map with the usage of a condition applied to the NDVI map 2022, as follows:

If NDVI <0.2 ►values = NODATA.

If NDVI <0.2 ►the same value applies.

**Fig. 4:**
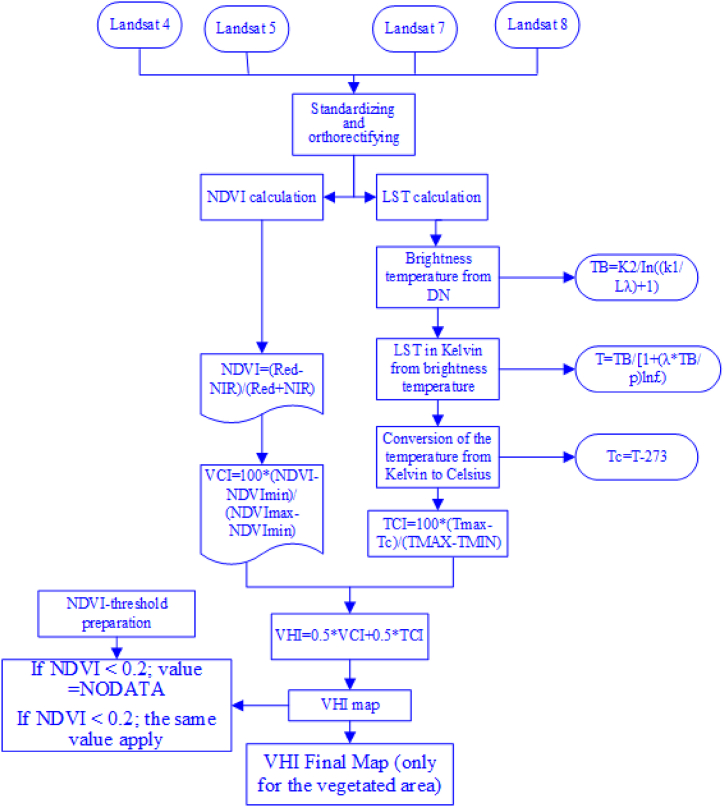
Methods flowchart for computing the vegetation health index (VHI).

Where the threshold value of the vegetated area is 0.2. The ultimate outcome was designated as “NDVI-threshold.” The final step would be to combine the VHI and the “NDVI-threshold” to produce a new VHI map that only shows vegetated areas. Fig. 4 shows the approach flowchart for the drought monitoring methods, which were used for the study period of 1993-2022to produce the drought condition (TCI, VCI, and VHI).

### 2.10 Summary of the research methodology

**Fig. 5:**
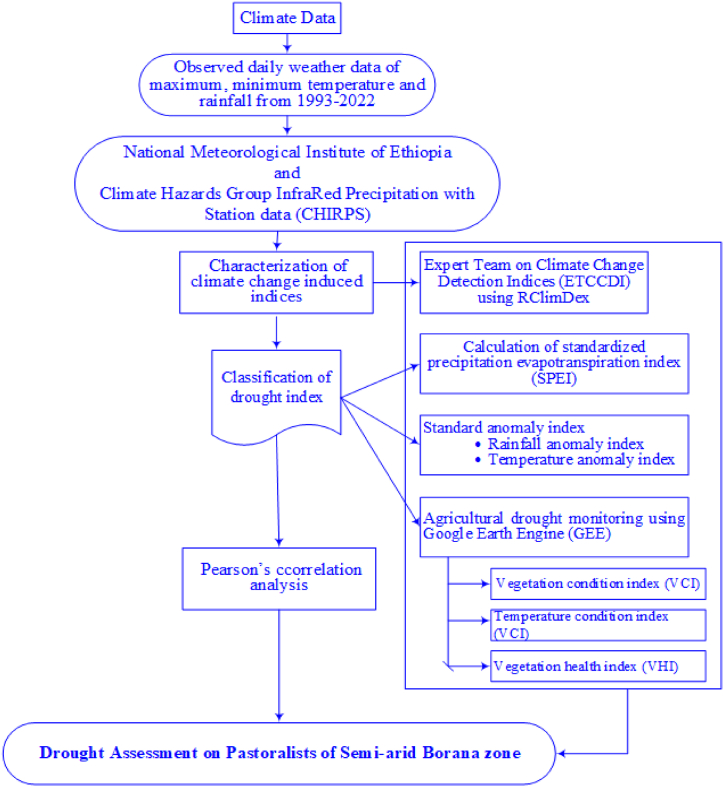
Summary of the research methodology.

The conceptual framework is developed for agricultural drought monitoring under climate change. Agricultural livelihoods are highly vulnerable to the impacts of drought. This vulnerability can be better understood through a comprehensive assessment that examines agrometeorological, biophysical, and socioeconomic variables. Fig. 5 shows the conceptual framework which can support the aims and objective of climate extreme indices and agricultural drought impacts assessment in semi-arid Borana zone.

## 3 Results and discussions

### 3.1 Climate change extreme indices across semi-arid areas of Borana zone

#### 3.1.1 Rainfall indices

Table 5 displays the long-standing trends of cumulative rainfall indices in different districts of mainly pastoralist areas in the Borana zone. Accordingly, the frequency of days with heavy precipitation (R10) was decreasing and Sen's slope per year in Dilo, Dire, Dubluk, Moyale, and Yabelo districts were −0.95, −2.20, −0.20, −0.12, and −0.210, respectively. This implies that, as the precipitation decreases the probability of dry spell increases causing shortages of pasture and water leading to substantial loss of livestock annually in the zone. Climate change and extreme weather have negative impacts on the livelihood of Borana pastoralists [31,65]. Similarly, Desta & Coppock [66] reported that between 1980 and 2000 the pastoralists in Borana zone experienced three major droughts that caused 35–67 % of livestock loss, which was worth hundreds millions dollars of USD.

**Table 5:** Trend analysis on rainfall indices the study area from 1993 to 2022.

Location		R10	R20	SDII	CDD	CWD	Rx1day	Rx5day	R95p	R99p	PRCPTOT
Dhaso	MK	1.11	1.86	1.86	−0.73	0.00	−2.23∗	2.23∗	2.31∗	1.81	−1.74
	Slp	0.138	0.000	0.030	−0.357	0.000	−0.671	0.828	3.392∗∗∗	0.182	−6.607∗∗∗
Dilo	MK	0–1.24	1.66	0.88	−0.82	0.38	1.18	1.22	0.29	1.85	−1.03
	Slp	−0.095	0.000	0.016	−0.467	0.067	0.506	0.975	0.524	0.000	−2.720∗∗
Dire	MK	2.10∗	1.39	1.76	1.18	−3.34∗∗∗	−1.31	2.68	1.33	1.48	0.39
	Slp	−2.20∗	0.077	0.055	0.444	−0.222	−1.560	1.891	4.400∗∗∗	−2.78∗∗	1.886
Dubluk	MK	0.67	1.79	1.74	−1.21	−0.39	−2.31∗∗	−2.04∗	2.12∗	−1.58	−1.67
	Slp	−0.200	0.000	0.033	−0.667	−0.077	−0.632	−0.845	3.600∗∗∗	1.450	−5.563∗∗∗
Guchi	MK	1.11	1.86	1.86	−0.79	0.00	−2.23∗∗	−2.23∗	−2.31∗	1.81	1.74
	Slp	0.138	0.000	0.030	−0.474	0.000	−0.671	−0.828	−3.392∗∗∗	0.182	−6.607∗∗∗
Moyale	MK	0.75	1.79	1.74	−1.21	−0.39	4.21∗∗	−2.75∗∗	−2.12∗	1.58	−1.67
	Slp	−0.120	0.000	0.033	−0.667	−0.077	0.842	−0.845	−3.600∗∗∗	1.450	−5.563∗∗∗
Taltale	MK	2.38∗	2.18∗	2.42∗	−0.13	0.54	1.78	−2.16∗	2.31∗	−2.0∗	−1.74
	Slp	0.333	0.158	0.057	−0.063	0.083	0.790	−1.25	7.808∗∗∗	3.12∗∗∗	−10.257∗∗∗
Wachile	MK	0.95	1.79	1.74	−1.21	−0.39	−2.31∗∗	2.04∗	2.12∗	1.58	−1.67
	Slp	0.100	0.000	0.033	−0.667	−0.077	−0.632	0.845	3.600∗∗∗	1.450	−5.563∗∗∗
Yabelo	MK	0.95	1.79	1.74	−1.41	−0.39	3.31∗∗	2.04∗	−2.12∗	1.58	−1.67
	Slp	−0.210	0.000	0.033	−0.667	−0.077	0.632	0.645	−3.500∗∗∗	1.450	−4.363∗∗∗

MK, Mann-Kendall trend test, slp; sen's slope (change/years).

Significance values: ‘∗∗∗’ 0.001 ‘∗∗’ 0.01 and ‘∗’ 0.05.

The study districts experienced significant decline in the number of consecutive dry days (CDD) but simple precipitation intensity index (SDII), maximum 5-day precipitation quantity (Rx5days), frequency of very heavy precipitation days (R20), very wet days (R95p), and extremely wet days (R99p). A decline by CDD may relieve burden on water supplies, increasing the amount of water available for agriculture, cattle, and human use. In the districts of Dire, Guchi, Wachele, and Moyale, the trend analysis showed a significant decline in the number of consecutive wet days (CWD), maximum 1-day precipitation amount (Rx1days), maximum 5-day precipitation amount (Rx5days), and very wet days (R95p) (Table 5). At 1 % and 5 % confidence levels, the corresponding factors of reduction were determined to be −3.34, −2.23, −2.31, and −2.75. The production of crops in the semiarid and arid portions of the agropastoral area in the Borana zone of Ethiopia may benefit from this reduction in the maximum 1-day precipitation quantity as it can lessen the likelihood of flash floods and soil erosion. These findings align with reports of IPCC (2023), which summarized that the frequency and intensity of extreme climate events, like heat waves, flooding, and droughts have been increasing and portended to increase in the future due to anthropogenic activities.

The majority of districts in the Borana zone experienced a minor rise in extremely wet days (R99p), except for Dire and Taltale, where a considerable decrease was noted at 1 % and 5 % confidence levels. The annual and monthly total precipitation (PRCPTOT) decreased in Dhaso (−6.6), Dilo (−2.72), Dubluk (−5.56), Guchi (−6.607), Moyale (−5.563), and Tatlate (−10.257) districts of Borana zone (Table 5). The amount of decrease varied depending on the location; Dhaso and Guchi showed the biggest decrease (−6.607), while Dilo showed the smallest decrease (−2.72). Drought brought on by lower precipitation levels can harm agriculture, food security, and the availability of drinking water. In addition, a drought may make wildfires and other natural calamities more likely. Reduced precipitation can have effects that go beyond human systems, influencing the health of the soil, the populations of plants and animals, and the general functioning of ecosystems.

##### 3.1.1.1 Number of heavy rain days (R10) and consecutive dry days (CDD)

**Fig. 6:**
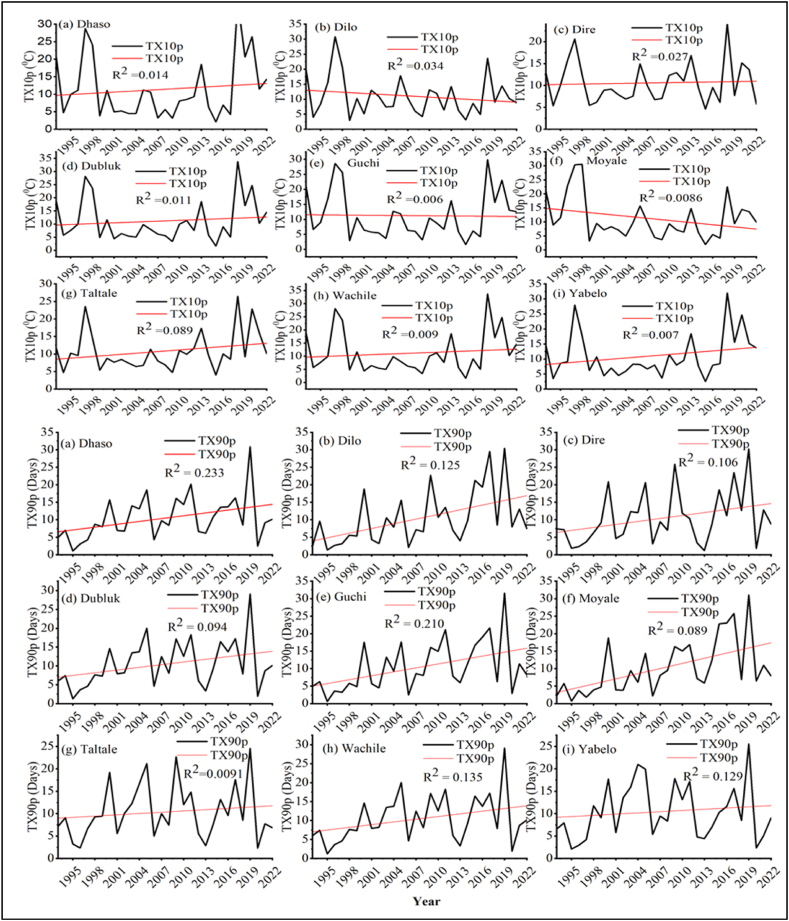
Number of heavy rain day (R10) and consecutive dry days (CDD) in the study area a) Dhaso, b) Dilo, c) Dire, d) Dubluk, e) Guchi, f) Moyale, g) Taltale, h) Wachile, i) Yabelo.

A variable and inconsistent pattern of upward and downward trends in the number of heavy rain days (R10mm) was observed in the districts of semi-arid Borana zone (Fig. 6 [a-i]). The number of heavy rain days showed a slightly increasing trend, but the trend was statistically non-significant. The result was in line with the findings of Mekasha et al. [67] reported that, inconsistencies and lack of statistical significance in the extreme rainfall indices in three Ethiopian eco-environments. Analysis of extreme consecutive dry days (CDD) revealed increase in Dhaso, Dilo, Dire, Guchi, Taltale, and Yabelo during the study period (1993–2022). The years 2003 and 2018 had the highest and lowest totals of consecutive dry days in Guchi and Taltale districts, respectively. This result is similar to findings of Teshome and Zhang [22] who reported an increasing trend of CDD at Kombolcha, Northern Ethiopia. Similar to the current results, Mohammed et al. [68] reported a significant increasing trend in CDD in the northwestern (Guba station) and eastern central (Mekane Selam station) parts of the Upper Blue Nile Basin of Ethiopia.

##### 3.1.1.2 Very wet days (R95p) and extremely wet days (R99p)

**Fig. 7:**
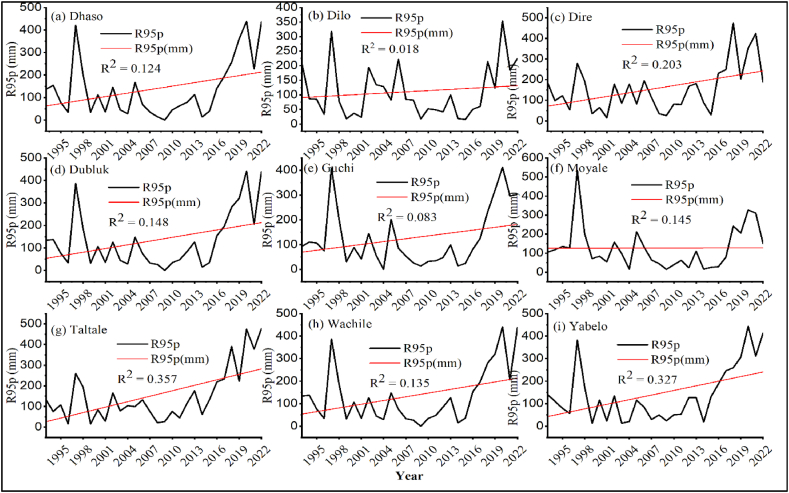
Very wet days (R95p) of different stations for (a) Dhaso, (b) Dilo, (c) Dire, (d) Dubluk, (e) Guchi, (f) Moyale, (g) Taltale, (h) Wachile, (i) Yabelo.

**Fig. 8:**
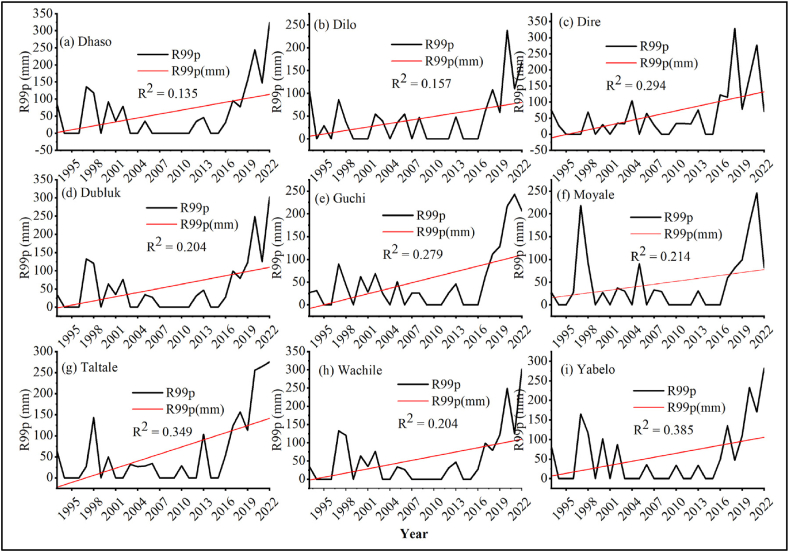
Extremely wet days (R99p) of different stations for (a) Dhaso, (b) Dilo, (c) Dire, (d) Dubluk, (e) Guchi, (f) Moyale, (g) Taltale, (h) Wachile, (i) Yabelo.

The result revealed that wet days (R95p) and extremely wet days (R99p) were observed in all nine districts of the study area (Fig. 7, Fig. 8 [a-i]). In the study area, the intensity of precipitation on very and extremely wet days (R95p and R99p) showed a non-significant increasing trend over the study period. The result was in line with the findings of Worku et al. [69] who reported upward trend for PRCPTOT, R95p, R99p and Rx5day indices. Similarly, Webster et al. [70] and Xie et al. [71] reported interannual monsoon precipitation extremes over Pakistan during the 2010 flooding and drought phases, which they attributed to large-scale atmospheric and oceanic forcings.

The occurrence of very wet and extremely wet days might adversely affect livestock health and productivity. Livestock disease epidemics can be exacerbated by prolonged wet circumstances, or by exposing animals to standing water or muddy environments [72]. Climate change is predicted to alter pest and disease outbreaks, increase the frequency and severity of floods and drought, and increase the likelihood of low yields, crop failure and livestock mortality [73,74].

##### 3.1.1.3 Total rainfall (PRCPTOT)

**Fig. 9:**
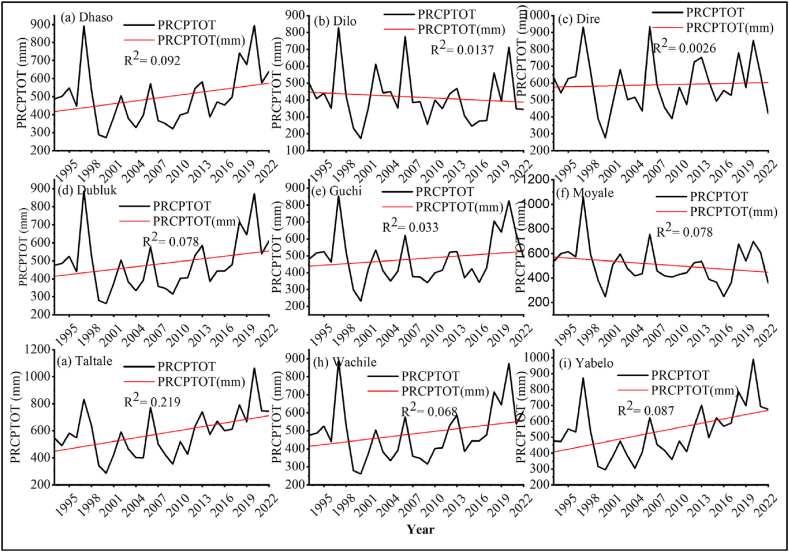
Annual total wet-day precipitation (PRCPTOT) in districts of (a) Dhaso, (b) Dilo, (c) Dire, (d) Dubluk, (e) Guchi, (f) Moyale, (g) Taltale, (h) Wachile, and (i) Yabelo.

The PRCPTOT (>1 mm/days) of Borana zone differed significantly between agroecological zones. The annual or monthly total rainfall (PRCPTOT) in the districts of Dhaso, Dilo, Dire, Dubluk, Guchi, Moyale, Taltale, Wachele, and Yabelo, were recorded below 0.050 mm/year (*p* < 0.05), which indicates the presence of severe drought conditions (Fig. 9 [a-i]). Precipitation is significant for agricultural activities in semi-arid and dry regions, and its variability can cause crop failure, desertification, loss of biodiversity and soil erosion. Previous studies noted continuum of rainfall intensity at different locations. In some places, global warming causes extreme rainfall intensities that cause flooding [75] whereas in other regions rainfall scarcity leads to drought in recent decades [76]. Similar studies also showed the absence of uniform temporal and spatial rainfall patterns [30,77]. Extended dry spells, decreased length of growing period and total lack of rainfall were perceived by local farmers as agro-meteorological variables with severe consequences on agricultural productivity [78].

#### 3.1.2 Extreme temperature variation in semi-arid areas of Borana zone

In this study, trend analysis of tropical nights (nights with a temperature of over 20 °C) in Dhaso, Dilo, Dire, and Moyale districts increased by factor of 2.54, 3.50, 2.43, and 2.93, respectively (Table 6). These results are in agreement with the findings of Erlat and Türkeş [79] that indicated that the frequency of tropical nights showed increasing trend and a continually growing tendency in most stations (87–92) after 1985. Cantos et al. [80] also reported increase in the frequency, length, and intensity of tropical nights in Valencia and Murcia regions of Spain's Mediterranean coast between 1950 and 2014.

**Table 6:** Trend analysis on extreme temperature indices from 1993 to 2022 for districts of Borana zone.

Location		TR20	CSDI	WSDI	SU25	TNx	TNn	TN10p	TN90p	TXx	TXn	TX10p	TX90p	DTR
Dhaso	Mk	2.54∗∗	−2.36∗∗	0.95	−0.54	1.18	−1.52	−2.36∗	3.39∗∗	1.66	−0.73	0.43	1.89	−1.43
	Slp	0.143	−0.100	0.000	0.000	0.018	−0.03	−0.286	0.37	0.014	−0.01	0.075	0.231	−0.014
Dilo	Mk	0.54	0.10	0.95	−0.05	2.94∗∗	−0.46	2.96∗∗	1.12	−1.46	−0.042	0.14	1.75	−1.87
	Slp	3.50∗∗	−2.03∗	0.000	0.000	0.059	0.000	−0.105	0.352	0.015	−0.042	0.015	0.245	−0.016
Dire	Mk	0.90	−0.73	0.21	−2.77∗∗	2.59∗∗	−0.79	−0.81	3.75∗∗	1.11	−0.39	0.14	1.75	−2.77∗∗
	Slp	2.43∗∗	0.000	0.000	−0.027	0.048	−0.017	−2.81∗∗	0.467	0.013	−0.007	0.015	0.245	−0.027
Dubluk	Mk	1.75	−1.01	0.00	−0.18	1.61	−0.30	−2.14∗	3.32∗∗	0.52	0–0.43	0.57	2.05∗	−1.66
	Slp	0.000	0.000	0.000	−0.091	0.030	−0.003	−0.293	0.380	0.007	−0.012	0.069	0.225	−0.016
Guchi	Mk	2.7	−0.97	1.82	0.96	1.70	−0.89	−2.75∗∗	1.59	1.21	0.32	−0.11	2.57∗∗	−0.39
	Slp	0.071	−0.286	0.000	0.333	0.030	−0.021	−0.305	0.486	0.013	0.007	−0.018	0.329	−0.004
Moyale	Mk	1.75	−2.01∗	0.00	−0.18	1.61	−0.30	−2.14∗	3.32∗∗	0.52	−0.43	0.57	2.05∗	−1.66
	Slp	0.000	0.000	0.000	−0.09	0.03	−0.003	−0.293	0.380	0.007	−0.012	0.069	0.225	−0.016
Taltalle	Mk	2.93∗∗	−0.61	−0.15	−0.45	1.75	−1.61	−0.96	2.27∗	1.11	0–0.71	1.18	0.75	−3.04∗∗
	Slp	0.895	0.000	0.000	0.000	0.03	−0.023	−0.142	0.243	0.014	−0.022	0.134	0.076	−0.029
Wachile	Mk	1.75	−1.01	0.00	−0.18	1.61	−0.30	−2.14∗	3.32∗∗	0.52	−0.43	0.57	2.05∗	−1.66
	Slp	0.000	0.000	0.000	−0.09	0.03	−0.003	−0.293	0.380	0.007	−0.043	0.069	0.225	-.016
Yabelo	Mk	1.75	−1.01	0.00	−0.18	1.61	−0.30	−2.14∗	3.32∗∗	0.52	−0.43	0.57	2.05∗	−1.66
	Slp	0.000	0.000	0.000	−0.09	0.03	−0.003	−0.293	0.380	0.007	−0.012	0.069	0.225	−0.016

MK, Mann-Kendall trend test, Slp; sen's slope (change/years).

Significance values: ‘∗∗∗’ 0.001 ‘∗∗’ 0.01 and ‘∗’ 0.05.

According to the MK-trend analysis, cold spell duration index (CSDI) dramatically reduced in Dhaso, Dilo, and Moyale, with values of −2.36, −2.03, and −2.0, respectively. However, the warm spell duration index (WSDI), monthly or yearly maximum of daily minimum temperature (TNx), warm nights (TN90p), and warm days (TX90p) showed increases across all districts (Table 6). According to the findings of Wubaye et al. [30], warm nights (TN90p) and days (TX90p) were increasing in several stations across Ethiopia. The WSDI, which measures the duration of warm spells, are characterized as a run of days with a daily maximum temperature higher than the 90th percentile of the baseline has also increased. The results in the current study and previous studies (eg. ubaye et al., 2023) suggest that there have been more prolonged and frequent heatwaves in various parts of Ethiopia in recent years. However, the trend analysis of chilly evenings (TN10p), monthly or annual minimum of daily maximum temperature (TXn), and diurnal temperature range (DTR) decreased in Borana zone (Table 6). The findings indicated that DTR is changing on a regional rather than global scale.

##### 3.1.2.1 Tropical nights (TR20) and cold spell duration index (CSDI)

**Fig. 10:**
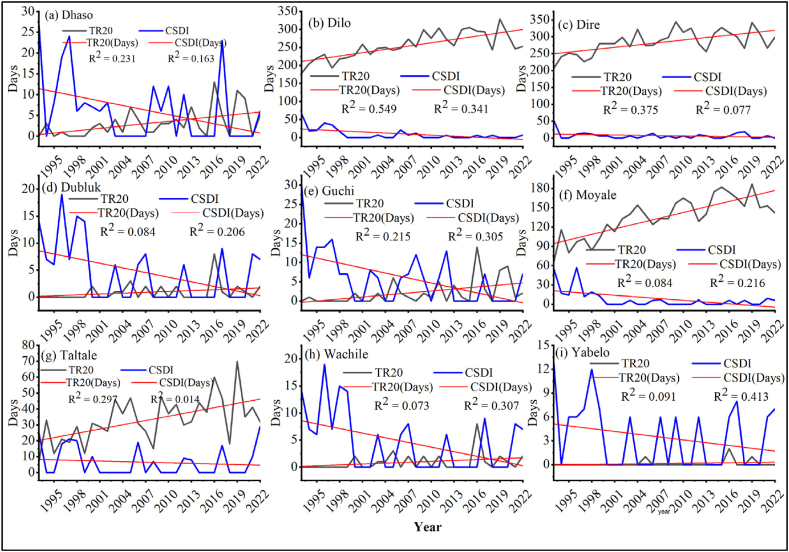
Tropical night (TR20) and Cold spell duration index (CSDI) (a) Dhaso, (b) Dilo, (c) Dire, (d) Dubluk, (e) Guchi, (f) Moyale, (g) Taltale, (h) Wachile, and (i)Yabelo.

Tropical nights (TR20) of the semi-arid areas of Borana zone illustrated increasing trend in all districts (Fig. 10[a-i]). Similar finding was reported by Teshome and Zhang [22], who showed an increasing trend in tropical nights (TR20) in a study undertaken over Ethiopia. Similarly, cold spell duration index (CSDI) also showed increasing trend in the study area. All districts of the Borana zone, which are mostly home to pastoralist communities experienced a statistically significant (P < 0.05) cold spell duration index (CSDI). The results revealed in this study are in agreement with the upward trend of cold spell duration reported in Gebrechorkos et al. [2].

##### 3.1.2.2 Cool days (TX10p) and warm days (TX90p)

**Fig. 11:**
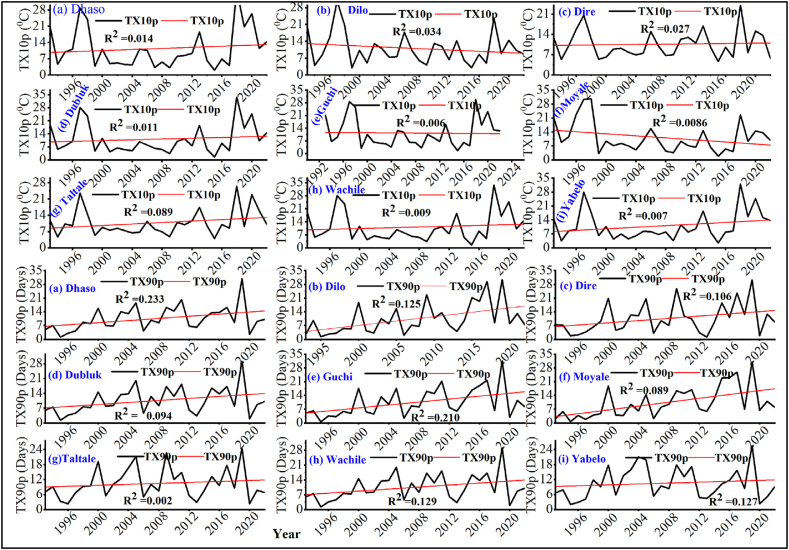
Cool days (TX10p) and warm days (TX90p) of temperature extreme indices for (a) Dhaso, (b) Dilo, (c) Dire, (d) Dubluk, (e) Guchi, (f) Moyale, (g) Taltale, (h) Wachile, and (i)Yabelo.

The monthly or yearly maximum of daily maximum temperatures (TX10p, and TX90p) increased in the districts of Borana zone (Fig. 11[a-i]). Similar trends of rising maximum temperature indices were reported throughout the African continent by various authors [[81], [82], [83], [84]]. Gebrechorkos and Hulsmann [85] also noted significant increases in warming indicator values and decreases in cold spell indicator values in Ethiopia. Accumulating literature collectively provides credence to the observation of shifting temperature patterns and the consequent effects on local biological processes and agricultural productivity [86]. Similarly, increasing trends of TX90p and TN90p reduce agricultural productivity of at various locations [87,88].

### 3.2 Classification drought indices

#### 3.2.1 Standardized precipitation-evapotranspiration index

**Fig. 12:**
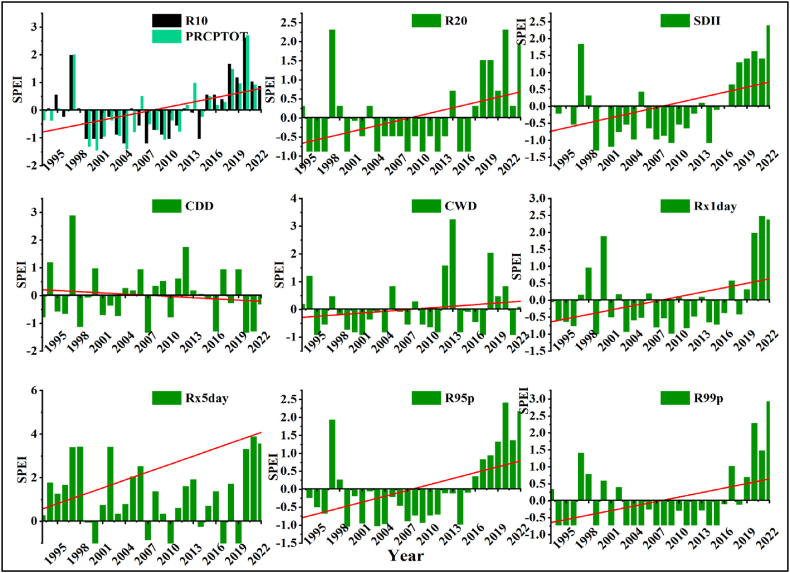
Standardized precipitation-evapotranspiration index (SPEI) over agroecological zones of Borana pastoralist for the period of 1993–2022.

Rainfall anomaly indices across semi-arid areas of Borana zone are shown in Fig. 12 [a-i]). Positive anomalies and extremely wet values were recorded for Rx5day from 2018 to 2022 in the area. This result aligns with the findings of Pita-Díaz and Ortega-Gaucin [89] who found increasing precipitation presented as a standardized anomaly of Rx5days per season in Zacatecas, Mexico.

The SPI of R10, R20, SDII, R1day, R95p, R99p, and PRCPTOT were extremely wet in the study area in 2019 (Fig. 12). This implies that the area could be associated with atmospheric phenomenon that resulted in high precipitation in the area. However, standardized precipitation-evapotranspiration indices of R10, R20, R95p, and R99p experienced moderate drought indices over the semi-arid areas of Borana one from 2001 to 2013.

#### 3.2.2 Standardized anomaly of changes in extreme temperature

**Fig. 13:**
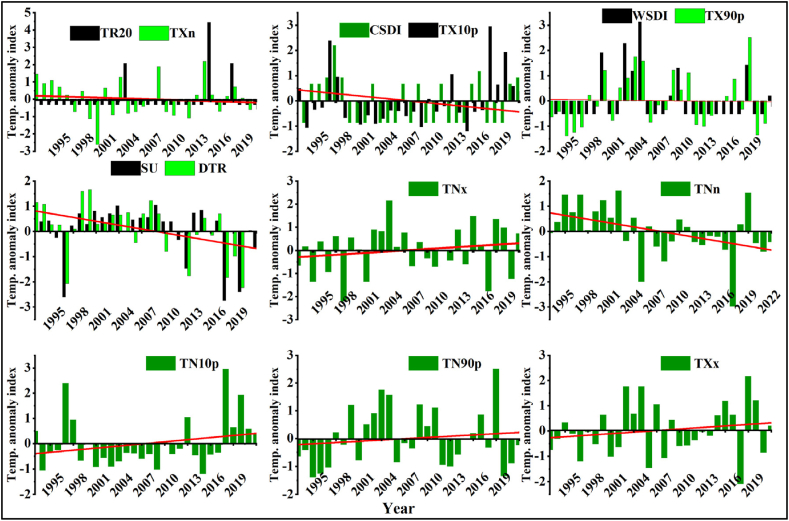
Standardized anomaly of temperature extreme indices over agroecological zones of Borana zone (1993–2022).

The maximum and minimum monthly and annual changes in extreme temperatures over semi-arid Borana zone from 1993 to 2022 are shown (Fig. 13[a-i]). The result indicates that, annual standardized anomalies reflect changes in extreme TR20 (2014), CSDI (1997 and 2017), TX10p (1997), WSDI (2003), TX90p (2017), TX10p (2018), TN90p and TXx (2019). During the aforementioned years, the anomalies were positive and extremely wet with values exceeding +2° in the study area. However, extremely dry and negative anomalies were observed with extreme changes in temperature of TXn (1999), SU and DTR (2018 and 2020), TNx (1998), and TNn (2017). Dry lands where pastoral and agropastoral population live account for approximately 60 % of the total land area of Ethiopia and 12 % of the population of the country [90]. In these area, climate extremes such as drought, high temperature are the main factors that cause a decrease in cattle productivity and threaten the livelihoods of farmers [91].

### 3.3 Correlation between rainfall and temperature extreme indices

Table 7 shows the correlation matrix of annual trend magnitudes for all 10 rainfall extreme indices in Borana zone. The results indicate positive correlations among precipitation indices such as R20, SDII, Rx1days, Rx5days, R95p, R99p, and PRCPTOT. This implies that high temperature could be associated with high precipitation as it can intensify the earth's water cycle. Additionally, the annual or monthly rainfall extremes (R95p, R99p, and PRCPTOT) showed a high level of statistical significance at 5 % and 1 % levels and positive correlations with R10, R20, and SDII.

**Table 7:** Pearson's product-moment correlation of matrix between rainfall extreme indices during the period of 1993–2022.

	R10	R20	SDII	CDD	CSD	Rx1days	Rx5days	R95p	R99p	PRCPTOT
R10	1	**0.992∗∗**	**0.843∗∗**	0.293	0.396	0.497	0.524	**0.866∗∗**	**0.691∗∗**	**0.869∗∗**
R20	–	1	**0.827∗∗**	0.291	0.421	0.490	0.525	**0.866∗∗**	**0.689∗∗**	**0.898∗∗**
SDII	–	–	1	0.193	0.136	**0.819∗∗**	**0.846∗∗**	**0.956∗∗**	**0.905∗∗**	**0.702∗∗**
CDD	–	–	–	1	0.084	−0.142	−0.079	0.102	−0.070	0.089
CSD	–	–	–	–	1	0.015	−0.014	0.224	0.112	0.548
Rx1days	–	–	–	–	–	1	**0.948∗∗**	**0.830∗∗**	**0.945∗∗**	0.491
RX5days	–	–	–	–	–	–	1	**0.835∗∗**	**0.921∗∗**	0.499
R95p	–	–	–	–	–	–	–	1	**0.949∗∗**	**0.805∗∗**
R99p	–	–	–	–	–	–	–	–	1	**0.670∗∗**
PRCPTOT	–	–	–	–	–	–	–	–	–	1

∗, ∗∗, Significant at 0.05 and 0.01 confidence level.

Correlation analysis among different extreme temperature indices, including TNX, TN90p, and TX90p are given in Table 8. The analysis revealed a positive and strong relationship between these indices and TR20. Conversely, CSDI and TN10p exhibited significantly negative correlation of −0.706 and −0.813, respectively. Similarly, TN90p and TX10p showed strongly negative correlations of −0.665 and −0.939, respectively, with TN10p and SU25 at confidence levels of 5 % and 1 %. These findings contribute to the growing body of evidence at different temporal and spatial scales, indicating a trend toward warming climatic conditions. It is anticipated that when normal temperatures rise, weather extremes will become more frequent and intense [92]. Despite multiple attempts to assess weather extremes, improved daily data archives are still needed to facilitate extensive studies in this area, particularly in developing countries [93].

**Table 8:** Pearson's product-moment correlation of matrix between temperature extreme indices during the period of 1993–2022.

	TR20	CSDI	WSDI	SU25	TNx	TNn	TN10p	TN90p	TXx	TXn	TX10p	TX90p
TR20	1	**−0.706**	0.386	0.430	**0.708**	−0.020	**−0.813**	**0.861**	0.487	0.099	−0.351	**0.698**
CSDI	–	1	−0.228	−0.321	−0.422	−0.158	**0.838**	−0.506	−0.290	−0.006	0.298	−0.417
WSDI	–	–	1	0.150	0.555	−0.199	−0.315	0.565	**0.730**	0.042	−0.110	**0.796**
SU25	–	–	–	1	0.250	−0.132	−0.540	0.269	0.285	0.248	**−0.939**	0.367
TNx	–	–	–	–	1	−0.236	−0.504	0.626	**0.740**	−0.035	−0.231	0.618
TNn	–	–	–	–	–	1	−0.122	−0.002	−0.377	0.189	0.218	−0.182
TN10p	–	–	–	–	–	–	1	**−0.665**	−0.437	−0.126	0.511	−0.573
TN90p	–	–	–	–	–	–	–	1	0.441	0.001	−0.130	**0.849**
TXx	–	–	–	–	–	–	–	–	1	0.195	−0.384	0.621
TXn	–	–	–	–	–	–	–	–	–	1	−0.308	0.088
TX10p	–	–	–	–	–	–	–	–	–	–	1	−0.296
TX90p	–	–	–	–	–	–	–	–	–	–	–	1

∗, ∗∗,Significant at 0.05 and 0.01 confidence level. The values **in bold** are significant at 1 % and 5 % confidence levels.

### 3.4 Agricultural drought monitoring using Google Earth Engine

The agricultural drought in Borana zone of southern Ethiopia is also computed using vegetation health index (VHI). Google Earth Engine (GEE)is used to process, derive, and map each of those agricultural and meteorological drought indicators.

#### 3.4.1 Vegetation condition index (VCI)

**Fig. 14:**
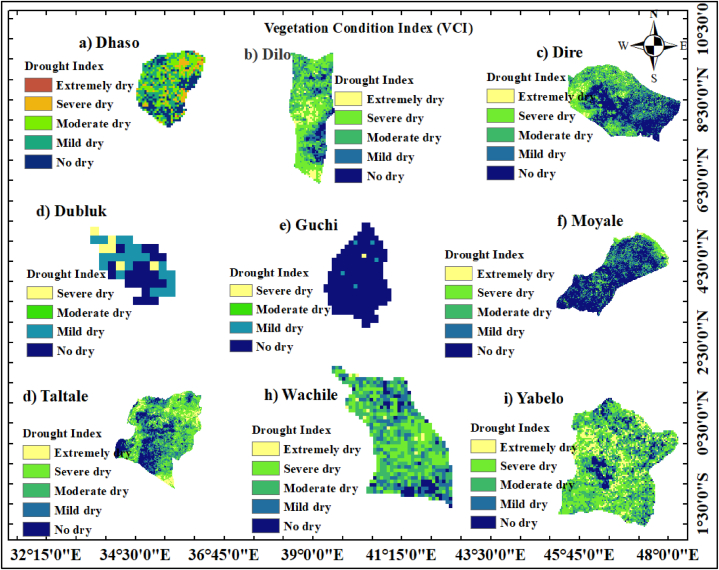
Annual vegetation condition index of 1993–2022 years for (a) Dhaso, (b) Dilo, (c) Dire, (d) Dubluk, (e) Guchi, (f) Moyale, (g) Taltale, (h) Wachile, and (i)Yabelo.

The annual VCI values obtained during 1993–2022 fell into four classes: extremely dry (<10), severely dry (11–20), moderately dry (21–30), mildly dry (31–40), no dry (>40) (Fig. 14[a-i]). The result of the annual VCI's spatial-temporal distribution indicates that vegetation decreased gradually over time and reached the extreme drought condition in semi-arid Borana zone. According to the spatial-temporal analysis of VCI, practically the districts of Dhaso, Dilo, Taltale, and Yabelo experienced extreme drought. Additionally, the districts of Dilo, Wachile, and Yabelo had a greater concentration of areas affected by severe drought, suggesting a reduced quantity of vegetated land and a greater vulnerability to areas susceptible to drought. Data from remote sensing are being used more to look into the temporal and spatial drought conditions related impacts on natural resources management. This result was in line with the findings of Vogt et al. [94] which detailed early attempts to monitor drought using remote sensing data. Ji and Peters [95] used NDVI to study the response of vegetation to drought condition. Thus, for countries with dry to semi-arid climates, getting such data is becoming increasingly crucial [96].

Regarding moisture and wet conditions, the Dire, Guchi and Moyale pastoralist areas of the Borana zone experienced favourable wet conditions. This implies that both vegetation dynamics and soil moisture levels were adequate, supporting nutrient uptake for crops or grasses that are palatable for livestock feed. According to Bhuiyan [64], favourable weather offers optimal moisture condition, and plant with high VCI values is healthy and unstressed. These areas were not affected by drought conditions, as indicated by the spatial-temporal map of the study area (Fig. 14). This findings align with the findings of Tsiros et al. [56], which demonstrated optimal weather conditions lead to maximum vegetation development due to enhanced utilization of ecosystem resources, including increased soil nutrient uptake.

#### 3.4.2 Temperature condition index (TCI)

**Fig. 15:**
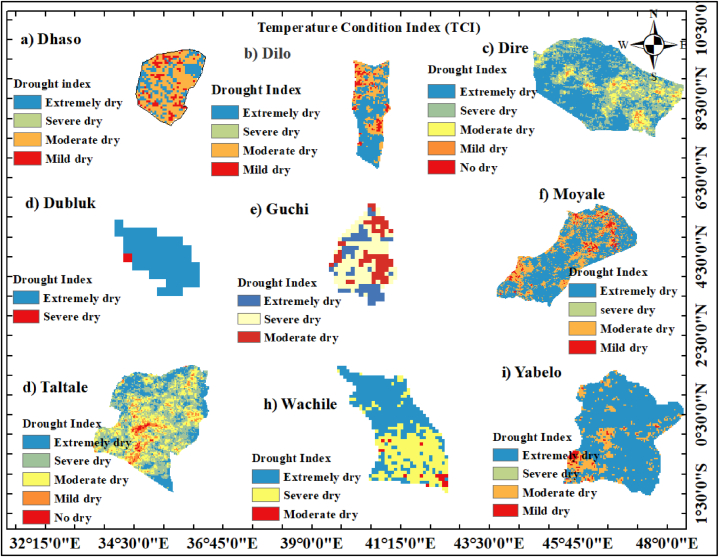
Annual temperature condition index from 1993 to 2022 years for (a) Dhaso, (b) Dilo, (c) Dire, (d) Dubluk, (e) Guchi, (f) Moyale, (g) Taltale, (h)Wachile, and (i)Yabelo.

The temperature condition index (TCI) is often used in studies evaluating droughts since temperature has a significant role in predicting the onset of drought. Fig. 15[a-i] shows the four classifications into which the yearly TCI for the years 1993–2022: extremely dry (<10), severely dry (11–20), moderately dry (21–30), mildly dry (31–40), and no dry (>40). The result shows that, TCI values in Dire, Dubluk, Moyale, and Yabelo districts showed drought conditions. The result is in agreement with the findings of Thenkabail and Gamage [97], that links low results of temperature condition index (TCI) to very hot weather for specific time period. Previous studies showed that TCI index can determine the regional effects of droughts before the loss of plant masses by establishing relationship between surface temperature and soil moisture (Ghalhari et al., 2022).

Spatiotemporal analysis of the TCI for Dhaso, Dilo, and Guchi districts revealed a moderate concentration of drought from 1993 to 2022. The high values of TCI in the range of no drought (>40) class were revealed in Dhaso, Dilo and some portion of Guchi district. This findings is in agreement with the results of Ayad et al. [98] that showed high TCI (>40) results reflect favourable conditions for vegetation with minimum temperature.

#### 3.4.3 Vegetation health index (VHI)

**Fig. 16:**
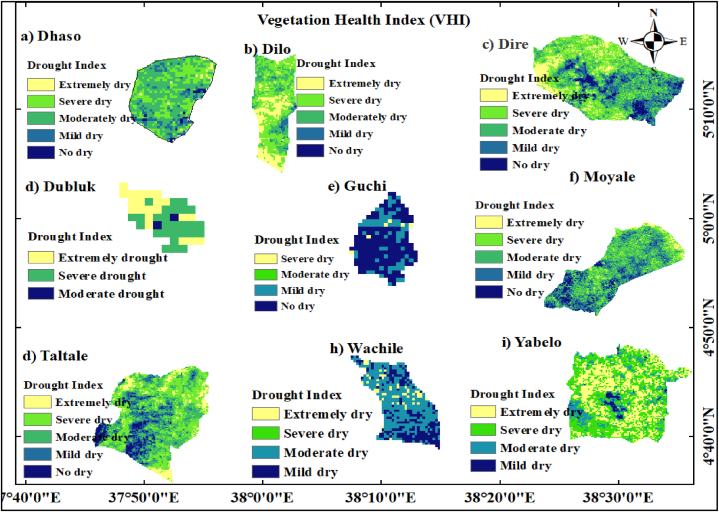
Annual vegetation health index from 1993 to 2022 years for (a) Dhaso, (b) Dilo, (c) Dire, (d) Dubluk, (e) Guchi, (f) Moyale, (g) Taltale, (h)Wachile, and (i)Yabelo.

The VHI values in the study area fell into four classes: i) extreme drought (<10), ii) severe drought (11–20), iii) moderate drought (21–30), iv) mild drought (31–40), and v) no drought (>40) (Fig. 16[a-i]). Annual VHI values in the districts of Dilo, Dire, Dubluk, Taltale, and Yabelo have been steadily growing over the study period, culminating in a significantly intensified VHI class of extreme drought conditions (<10). The VHI, as a monitoring tool allows one to virtually track the interaction between temperature, vegetation, and climatic influence in real-time [98]. In the Dilo, Dubluk, and Yabelo, there was a reduction in the distribution of good vegetation. Furthermore, the study area is covered by the severe drought with VHI class distribution (11–20) in the Dilo, Dubluk, and Yabelo districts. Rainfall in arid and semi-arid zones is erratic and unevenly distributed in space and time, which has an effect on the growth of plants in these environments due to seasonal and annual fluctuations [64]. Conversely, the healthy vegetation index was found in Guchi, Moyale, and Wachile districts that experienced mild drought (31–40) and no drought (>40) classes. According to Schmidt and Karnieli [99], desert ecosystem usually have a single rainy season when vegetation temporarily grow. This is followed by an extended period of drought that drastically lowers the amount of green vegetation in the area.

## 4 Conclusion

This study assessed the changes in agricultural drought and climatic extreme indices in Borana zone, Southern Ethiopia. The decline in the annual and monthly total precipitation in the majority of the districts implies that the area experiences uncertain rainfall condition, which is not favourable to agricultural production and livelihoods of pastoral and agropastoral communities of Borana zone. Half of the study districts had tropical night with recorded temperature that could cause heat stress, dehydration, exhaustion, heat stroke, decrease in plant production. Increased warm spells due to high maximum and minimum temperature, warm nights (TN90p), and warm days (TX90p) in the study area clearly indicate the area is highly challenged by climate change-induced extremes. Overall, Borana zone faces high risk of drought, which requires careful adaptation and mitigation strategies to sustain agricultural production and ecosystem productivity in the area. More accurate and precise climate information services and better investment on adaptation technologies are urgently needed.

## CRediT authorship contribution statement

**Girma Asefa Bogale:** Writing – original draft, Methodology, Data curation, Conceptualization, Software. **Asfaw Kebede Kassa:** Investigation, Visualization. **Mengistu Mengesha Maja:** Writing – review & editing, Validation.

## Data availability Statement

The data will be made available on reasonable request.

## Declaration of competing interest

The authors declare that they have no known competing financial interests or personal relationships that could have appeared to influence the work reported in this paper.
